# Evidence from community level inputs to improve quality of care for maternal and newborn health: interventions and findings

**DOI:** 10.1186/1742-4755-11-S2-S2

**Published:** 2014-09-04

**Authors:** Zohra S Lassi, Jai K Das, Rehana A Salam, Zulfiqar A Bhutta

**Affiliations:** 1 Division of Women & Child Health, Aga Khan University, Karachi, Pakistan; 2 Program for Global Pediatric Research, Hospital For Sick Children, Toronto

**Keywords:** Quality of care, community based interventions, maternal health, newborn health, community mobilization, support groups, outreach, human resource, training, community health worker

## Abstract

Annually around 40 million mothers give birth at home without any trained health worker. Consequently, most of the maternal and neonatal mortalities occur at the community level due to lack of good quality care during labour and birth. Interventions delivered at the community level have not only been advocated to improve access and coverage of essential interventions but also to reduce the existing disparities and reaching the hard to reach. In this paper, we have reviewed the effectiveness of care delivered through community level inputs for improving maternal and newborn health outcomes. We considered all available systematic reviews published before May 2013 on the pre-defined community level interventions and report findings from 43 systematic reviews.

Findings suggest that home visitation significantly improved antenatal care, tetanus immunization coverage, referral and early initiation of breast feeding with reductions in antenatal hospital admission, cesarean-section rates birth, maternal morbidity, neonatal mortality and perinatal mortality. Task shifting to midwives and community health workers has shown to significantly improve immunization uptake and breast feeding initiation with reductions in antenatal hospitalization, episiotomy, instrumental delivery and hospital stay. Training of traditional birth attendants as a part of community based intervention package has significant impact on referrals, early breast feeding, maternal morbidity, neonatal mortality, and perinatal mortality. Formation of community based support groups decreased maternal morbidity, neonatal mortality, perinatal mortality with improved referrals and early breast feeding rates. At community level, home visitation, community mobilization and training of community health workers and traditional birth attendants have the maximum potential to improve a range of maternal and newborn health outcomes. There is lack of data to establish effectiveness of outreach services, mass media campaigns and community education as standalone interventions. Future efforts should be concerted on increasing the availability and training of the community based skilled health workers especially in resource limited settings where the highest burden exists with limited resources to mobilize.

## Background

Annually around 40 million mothers give birth at home without any trained health worker [1]. Consequently, most of the maternal, perinatal and neonatal morbidities and mortalities occur at the community level due to lack of good quality care during labour and birth. The poorest and fragile countries have the highest neonatal mortality rates and preventable deaths depicting the existing inequities [1,2]. The causes are multi-factorial, ranging from socio-economic aspects of poverty; poor health status of women; lack of autonomy and decision making authority; and illiteracy to health system related factors like poor antenatal and obstetric care; absence of trained birth attendant; inadequate referral system; lack of transportation facilities; and poor linkages between health centers and communities [1,3]. This burden could be averted by achieving universal coverage in skilled birth attendance and providing good quality care for all births. However due to paucity of trained human resource professionals in first-level health services and the reduced awareness of and accessibility to services for the deprived and marginalized populations, these are not accessible to the ones in need [1,4].

Community based delivery is now widely recognized as an important strategy to deliver key maternal and child survival interventions [5-10]. It has been instrumental in scaling up coverage of certain interventions, such as immunization, oral rehydration therapy for diarrhea, tuberculosis treatment and family planning initiatives. Interventions delivered at the community level have not only been advocated to improve access and coverage of essential interventions but also to reduce the existing disparities and reaching the hard to reach. Inputs at the community level involve programs based on training and consequent task shifting from healthcare personnel to mid-level health care personnel or lay individuals for local implementation at home, village or any defined community group. They focus on resources such as local knowledge, volunteers’ time, community confidence and trust as channels for delivery. Community platforms can be used to deliver a full spectrum of promotive, preventive, and curative interventions including provision of basic antenatal (ANC), natal and postnatal care (PNC); preventive essential newborn care; breastfeeding counseling; management and referral of sick newborns; skills development in behavior change communication; and community mobilization strategies to promote birth and newborn care preparedness. These programs do not substitute for a formal health system, but provide a channel to reach far flung areas with knowledge, commodities and skills, thus attempting to reduce existing inequities in healthcare access and utilization. In this paper, we have reviewed the effectiveness of care delivered through community level inputs for improving maternal newborn health (MNH) outcomes. For this review, we have broadly categorized these interventions into four categories: outreach services (including home visitation and referrals); task shifting; training; and formation of support groups for community mobilization.

## Community level characteristics

### Outreach services and home visitation

Home-based strategies to support optimal maternal and neonatal care practices have emerged in the last decade to complement facility-based care and promote universal access to and utilization of health services with a specific focus on low- and middle- income countries (LMICs).These services mainly include ANC, skilled birth attendance and early PNC. Such programs involve standardized or individualized interventions for additional support provided during home visits, regular ANC visits, and/or by telephone throughout pregnancy by multidisciplinary teams of health professionals and trained lay workers. The major benefit of these programs is that the service is brought to the remote population in their own homes and allow care providers to deliver a more tailored health care service.

### Task shifting

Task shifting for human resource management involves substituting specialized personnel with healthcare workers that are lesser trained but can perform some aspects of their tasks. A range of both skilled and semi-skilled health workers can play a major role in MNH service delivery. Community health workers (CHW) and traditional birth attendants (TBA) are considered semi-skilled workers, while mid-level health workers (MLHW) such as nurses, midwives, associate clinicians, medical assistants and nurse auxiliaries are skilled workers certified for their work. Health service delivery through these skilled and semi-skilled healthcare workers has been practiced in both high-income countries (HIC) and LMIC all over the world for the past several decades. Evidence suggests that they can contribute significantly in improving health of the populations. More recently, due to the growing human resource crisis especially in LMICs, task shifting has re-emerged for extending services to hard-to-reach groups by substituting health professionals for a range of tasks [11-14]. Countries in south Asia and Africa have made a particular effort in recent years to reduce maternal and neonatal mortality and morbidity through deploying CHW [15,16]. The role of midwives and TBA in delivering MNH services has also received growing attention in the last few years, and a number of publications have described their role and documented the effects of such programs [17,18]. However, less attention has been given to assess the effectiveness of MLHW in delivering and improving health care delivery [19].

### Human resource training

Globally there is a growing shortage of 7.2 million healthcare workers and around 90% of all maternal deaths and 80% of still births occur in countries that lack trained healthcare workforce [20]. Although skills-mix imbalances persist, advanced practitioners, midwives, nurses and auxiliaries are still insufficiently used in many settings*.* Many LMIC are increasingly facing difficulties in producing, recruiting and retaining health professionals as they tend to migrate to wealthier countries due to low salaries, poor working conditions, lack of supervision, low morale and motivation and lack of infrastructure [9,21,22]. To overcome the failure of providing birthing women with skilled attendance, poor countries are now investing on training MLHW to at least provide them with some sort of assistance instead of none at all. In countries like Malawi, Bangladesh, Pakistan and Guatemala, training and close supervision of TBA have been evaluated to improve MNH outcomes and have shown some potential in reducing harmful practices during delivery and childbirth and improving MNH outcomes [23,24]. These training courses include short and structured approach to equip lay workers with lifesaving tools like Newborn Life Support (NLS), Neonatal Resuscitation Program (NRP) and Essential Newborn Care (ENC) but might vary in origin, scope and target audience. Besides additional training of outreach workers, human resource training also includes conferences, lectures, workshops, group education, seminars and symposia.

### Community mobilization and support groups

Soon after the Alma-Ata Declaration, it was recognized that community participation was important for the provision of local health services and for delivering interventions at the community level. Since then, it has been advocated to build its links with improving MNH [25]. Community support groups and women’s groups are now increasingly becoming a core component of community service package comprising of community representatives for health promotion. The objective is to enable the community to provide support to pregnant women and families throughout pregnancy and delivery. Communities are encouraged to identify key barriers to care and select the most appropriate interventions for their situation. Community mobilization also helps educate about available services, identification of danger signs during pregnancy, and the importance of seeking care from skilled healthcare worker during obstetric and newborn emergencies. A range of promotive messages, quality care and scale up coverage for MNH can be delivered through community workers and women’s groups [26].

**Figure 1:**
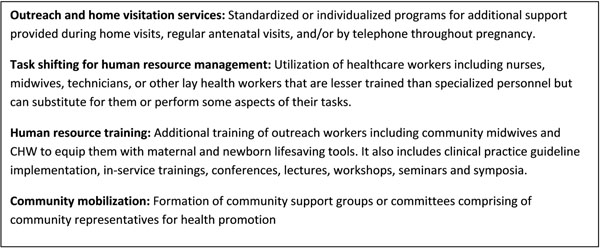
Components of community level interventions

In this review, we aim to systematically review and summarize the available evidence from relevant systematic reviews on the impact of the outlined community level inputs Figure 1). to improve the quality of care for women and newborns.

## Methods

We considered all available systematic reviews published before May 2013 on the pre-defined community level interventions as outlined in our conceptual framework [27]. A separate search strategy was developed for each component using broad keywords, medical subject heading (MeSH), and free text terms: [(Community OR home OR “home visit*” OR outreach OR “task shift*” OR “human resource” OR “in-service” OR training OR mobilization OR “support group*”OR “women’s group” OR “health worker*” OR “community health aides” OR “primary health care” OR “community health worker*” OR “lay health worker*” OR “mid-level health worker*” OR “community based interventions”) AND (health OR maternal OR mother OR child OR newborn OR neonat*)]

Our priority was to select existing systematic reviews of randomized or non-randomized controlled trials, which fully or partly address the a priori defined community delivered interventions for improving quality of care for MNH. We excluded reviews on home visits for prevention or screening for child abuse, maltreatment and childhood injury prevention as these were not included in the scope of our review. Search was conducted in the Cochrane library and PubMed and reviews that met the inclusion criteria were selected and data was abstracted by two authors on a standardized abstraction sheet. Quality assessment of the included reviews was done using Assessment of Multiple Systematic Reviews (AMSTAR) criteria [28] as detailed in paper 1 [27]. Any disagreement between the primary abstractors was resolved by the third author. For the pre-identified interventions, which did not specifically report MNH outcomes, we have reported the impacts on other health outcomes reported by the review authors. Estimates are reported as relative risks (RR), odds ratios (OR), risk differences (RD) or mean differences (MD) with 95% confidence intervals (CI) where available. For detailed methodology please refer to paper 1 of the series [27].

## Findings

**Figure 2:**
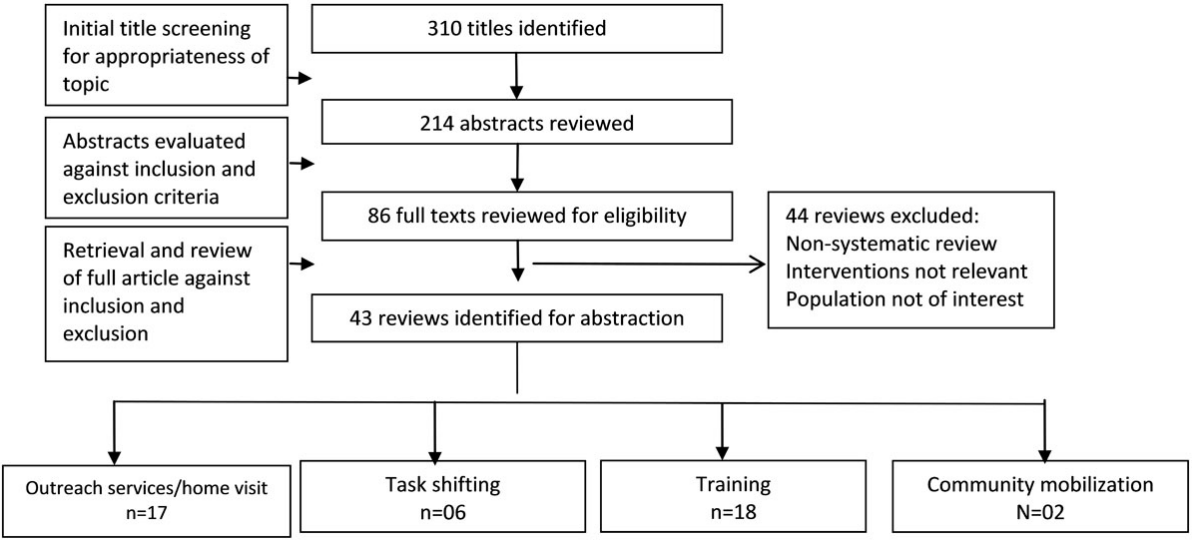
Search flow diagram

Our search yielded 310 potentially relevant review titles. Further screening of abstracts and full texts resulted in the inclusion of 43 eligible reviews: 17 for outreach services (home visitation and referrals), 6 for task shifting, 18 for human resource training and 2 for community mobilization (Figure 2). The overall quality of the reviews ranged from 3 to 11 with a median of 9 on the AMSTAR criteria.

### Outreach Services

We included 16 [29-44] reviews and 1 [45] overview of reviews pertaining to outreach and home visitation services with the median quality score of 7 on AMSTAR criteria. All reviews except one [33] reported MNH specific outcomes. The most commonly reported outcomes included maternal, newborn morbidity and mortality, immunization rates, breast feeding, referral, ANC and PNC utilization. Meta-analysis was conducted in six of the reviews. Reviews evaluating the impact of structured nurse- or midwife-based home visitation programs were from HIC while those focusing on service delivery through CHW were from LMIC. Table 1 summarizes the characteristics of the included reviews.

**Table 1:** Characteristics of the included reviews for outreach services, home visitation and referrals

Reviews (n=16)	Description of included interventions	Type of studies included (no)	Targeted health care providers	Outcome reported		Pooled data (Y/N)	Results
						
				Other outcomes	MNCH specific outcomes		
**Blondel 1995 **[29]	Two different types of home visits during pregnancy: (1) those offering social support to high-risk women; and (2) those providing medical care to women with complications.	RCT’s: 08	Nurses, family workers, midwives in HIC		Preterm delivery	Yes	1.0 (0.8-1.1)
					Hospital admission with complications		0.9 (0.7-1.2)

**Bull 2004 (Overview) **[45]	Home visiting is not a single or uniform intervention – it is a mechanism for the delivery of a variety of interventions directed at different outcomes. They may provide parent training/education, pyscho-social support to parents, infant stimulation, and infant and maternal health surveillance	Reviews: 09	Nurses, midwives or lay people within different professional bases in HIC		Pregnancy outcome	No	No impact
					Immunization rate		No impact
					Hospital admission		No impact
					Child injury		Positive impact
					Post natal depression		Positive impact

**Ciliska 2001 **[30]	Public health nursing interventions when carried out by the strategy of home visiting of clients in the pre- and postnatal period	20 studies RCT's: 8 CCT: 3 analytic cohort: 1	Nurses or midwives in HIC		Children’s mental development, mental health and physical growth, mother’s depression, maternal employment, education, nutrition and other health habits, and government cost saving.	No	No negative impacts reported in 12 strong articles
							Children mental and physical health improved
							No impacts on LBW, gestational age and neonatal morbidity and mortality

**Elkan 2000 **[31]	Home visiting program with at least one postnatal visit	102 papers with 86 home visiting programs	Nurses or midwives in HIC		Mental development score	Yes	0.17 (0.06-0.28)
					Motor development score		0.17(–0.03-0.38)
					IQ		0.32 (0.146-0.48)
					Weight		0.04 (–0.17-2.46)
					Height		0.04 ( –0.17-2.5)
					Immunization rate		1.40 (1.16-1.68)
					Use of acute care		0.73 (0.55-0.98)
					Hospital stay		1.63 (1.18-2.24)
					ER		0.77 (0.58-1.03)

**Gogia 2010 **[32]	Implementation by community health workers of safe delivery practices at home and proper care of the neonate immediately after birth, such as keeping the baby warm, providing neonatal resuscitation (if required) and initiating breastfeeding early.	RCT: 05	CHW in LIC		ANC visit >1	Yes	1.33 (1.20-1.47)
					Tetanus Toxoid (2 doses)		1.11 (1.04-1.18)
					Skilled care at birth		1.54 (0.81 - 2.93)
					Breastfeeding within 1 hour		3.35 (1.31-8.59)
					Clean cord care		1.70 (1.39-2.07)
					Delayed bathing		4.63 (2.29-9.37)
					Neonatal mortality		0.62 (0.44-0.87)
					Infant mortality		0.41 (0.30–0.57)
					**Neonatal cause-specific mortality due to:**		
					Sepsis		89.8% (78.6–101.0)
					Asphyxia		53.3% (23.8–82.8)
					Prematurity		38% (4.3–71.6)
					Hypothermia		100% (one-sided 95% CI not stated)

**Gruen 2003 **[33]	Specialist outreach clinics: defined as planned and regular visits by specialist-trained medical practitioners from a usual practice location (hospital or specialist center) to primary care or rural hospital settings.	RCT: 05 CBA: 02 ITS: 02	Primary healthcare practitioners and specialists	Adherence to treatment		Yes	0.63 (0.52-0.77)
				Patient and provider satisfaction			0.43 (0.29-0.62)
				Use of service			0.14 (0.05-0.32)

**Hodnett 2000 **[34]	Standardized or individualized programs of additional social support provided in either home visits, during regular antenatal clinic visits, and/or by telephone on several occasions during pregnancy.	RCT: 17	Multidisciplinary teams of health professionals specially trained lay workers, or combination of lay and professional workers.		Caesarean birth		0.87 (0.78-0.97)
					Gestational age less than 37 weeks at birth		0.92 (0.83-1.01)
					Birth weight less than 2500 gm		0.92 (0.83-1.03)
					Stillbirth/neonatal death		0.96 (0.74-1.26)
					Antenatal hospital admission		0.79 (0.68-0.92)
					Postnatal re-hospitalization		1.60 (0.80-3.21)
					Antenatal depression		0.77 (0.50, 1.19)
					Postnatal depression		0.85 (0.69-1.05)
					Less than highly satisfied with antenatal care		1.13 (0.76, 1.67)

**Hussein 2012 **[35]	Interventions included aimed to overcome delays in reaching the appropriate facility, which improved emergency referrals antenatally, during labour, or up to 42 d after delivery.	Total: 19 RCT: 04,controlled before after: 05,Cohort: 05	Community groups and TBA		Neonatal mortality	No	0.48 (0.34-0.68)
					Stillbirths		0.56 (0.32-0.96)

**Hussein 2012 **[36]	Refer pregnant and post-partum women suffering from an emergency obstetric complication or from home to basic-level health facilities (health centres) and from health centre to hospital (but not referral between hospitals) in LMIC	Total: 19 RCT: 04,controlled before after: 05,Cohort: 05	Community groups and TBA		Neonatal mortality	No	0.48 (0.34-0.68)
					Stillbirths		0.56 (0.32-0.96)

**Issel 2011 **[37]	Prenatal home visiting was defined as a nonmedical program or service focused on facilitating utilization of health or social services, provided in the home to pregnant women who were at high medical or social risk for adverse birth outcomes.	Total : 28 RCT: 14, descriptive: 2, retro cohort: 07, prospect cohort: 02, matched CC: 01, ecological : 01, static group: 01	Home visiting personnel not defined		PNC utilization	No	5/5 studies found significant improvement
					Gestational age		5/24 found a significant positive effect
					Birth weight		7/17 found a significant positive effect

**Kandrick 2000 **[38]	The home visitation program had to include at least one post natal home visits	11 RCT’s (9 meta-analyzed)	Nurses and midwives in HIC		Immunization uptake	Yes	1.17 (0.33-4.17) (Random)
							1.67 (1.29-2.15) (fixed)

**Lassi 2010 **[39]	Intervention packages that included additional training of outreach workers in maternal care during pregnancy, delivery and in the postpartum period; and routine newborn care.	18 cluster-randomized/quasi-randomized trials	outreach workers in LMIC		Maternal mortality	Yes	0.77 (0.59-1.02)
					Maternal morbidity		0.75 (0.61-0.92)
					Neonatal mortality		0.76 (0.68-0.84)
					Perinatal mortality		0.80 (0.71-0.91)
					Referral		1.4 (1.19-1.65)
					Early breast feeding		1.94 (1.56-2.42)

**Lonkhuijzen** 12 [44]	All types of facilities within easy reach of a medical facility that are designated for the lodging of pregnant women who await labour, with the purpose of the women being assisted by skilled attendants during delivery	None	Not applicable	Not applicable			

**McNaughton 2004 **[40]	Home-visiting interventions using professional nurses as home visitors.	13 reports	Nurses in HIC		Maternal newborn health status	No	Narrative (more than half of the studies were able to achieve their desired results)

**Peacock 2013 **[43]	Effect of paraprofessional home-visiting programs on developmental and health outcomes of young children from disadvantaged families.	21 studies	Paraprofessional home-visiting staff		Child abuse and neglect	No	3 out of 6 studies showed better outcomes
					Physical growth		5 out of 7 studies showed no significant improvement
					Hospitalization, illness and injuries		2 out of 6 studies showed better health outcomes
					Up-to date immunizations		1 study showed intervention group more likely to receive primary immunizations

**Pyone 2012 **[41]	Distance and transport cost related interventions	5 studies	Community		MMR (associated with distance)	No	7.4 (1.6 – 132.4)

**Vieira 2012 **[42]	Interventions to increase birth with skilled health personnel, in settings where TBAs were providers of childbirth care	6 observational studies	Skilled birth attendant		Obstetric mortality ratio	No	Deploying skilled health personnel and addressing financial barriers for users increased the use of skilled health personnel at birth
					Decrease in maternal deaths		
					Birth by a physician		
					Birth by C-Section		
					Increase in skilled birth attendance		

Home visits by CHW to improve neonatal health was associated with improved ANC (RR: 1.33, 95% CI: 1.20-1.47), tetanus immunization coverage(RR: 1.11, 95% CI: 1.04-1.18), breast feeding initiation within 1 hour (RR: 3.35, 95% CI: 1.31-8.59) and clean cord care (RR: 1.70, 95% CI: 1.39-2.07) [32]. Community based packages with an emphasis on provision of care through trained CHW via home visitation significantly improved maternal morbidity (RR: 0.75, 95% CI: 0.61-0.92), neonatal mortality (RR: 0.76 95% CI: 0.68-0.84), perinatal mortality (RR: 0.80, 95% CI: 0.71-0.91), referral (RR: 1.4, 95% CI: 1.19-1.65) and early breast feeding initiation (RR: 1.94, 95% CI: 1.56-2.42) [39]. A review evaluating the effectiveness of emergency obstetric referral interventions in LMIC showed that community based interventions for generating funds for transport reduced neonatal deaths in India (OR: 0.48, 95% CI: 0.34-0.68) while maternity waiting home interventions in sub-Saharan Africa reported reductions in stillbirths (OR: 0.56, 95% CI: 0.32–0.96) [35]. Another review to assess the effects of a maternity waiting facility on maternal and perinatal health did not find any trial for inclusion [44].

Nurse- or midwives- based home visit programs did not report any significant impact on birth outcomes, hospital admission for complications and neonatal morbidity and mortality. However, some positive impacts were reported on immunization rates (RR: 1.67, 95% CI: 1.29-2.15); child health outcomes including mental and physical health; and injury prevention [29,30,38,45]. Home visits with a specific focus on post natal visit was found to be associated with improved immunization rates (RR: 1.40, 95% CI: 1.16-1.68) with non-significant impacts on child’s mental and physical health [31]. Programs offering additional social support for pregnant women at high risk for preterm or low birth weight (LBW) delivery showed significant impacts on reducing the likelihood of antenatal hospital admission (RR: 0.79, 95% CI: 0.68-0.92) and cesarean birth (RR: 0.87, 95% CI: 0.78-0.97) when compared to routine care [34]. Specialist out-reach clinics did not have any impact on health outcomes but improved compliance to treatment (RR: 0.63, 95% CI: 0.52-0.77), patient-provider satisfaction (RR: 0.43, 95% CI: 0.29-0.62) and access [33].

### Task shifting

We included six [41,46-50] reviews pertaining to task shifting with a median score of 10 on AMSTAR criteria. Four reviews evaluating the impact of task shifting to CHW and midwives in LMIC reported MNH outcomes while the other two reviews from HIC focused on the impact of dietary counseling delivered through dietician versus nurses/doctors [50] and impact of nurses working as substitutes for primary care doctors [48]. Table 2 summarizes the characteristics of the included reviews.

**Table 2:** Characteristics of the included reviews for Human Resources-Task Shifting

Reviews (n=06)	Description of included interventions	Type of studies included (no)	Targeted health care providers	Outcome reported		Pooled data (Y/N)	Results
						
				Other outcomes	MNCH specific outcomes		
**Bhutta 2012 **[71]	Mid-level healthcare provider defined as those who have received less training than doctors but who perform aspects of doctors’ tasks.	RCT/cRCT: 52 ITS: 02 Case Control:01 Before After: 01	Nurse, midwives, auxillary nurse, auxillary nurse midwife, surgical technicians in both HIC and LMIC		**Wives versus doctors + midwives:**	Yes	
					Rate of performing c- section		0.92 (0.81-1.15)
					Postpartum hemorrhage		1.03 (0.82-1.29)
					Overall fetal or neonatal deaths		0.95 (0.69-1.30)
					Preterm births		0.87 (0.73-1.04)
					Admission to neonatal intensive care		1.03 (0.77-1.38)
					The use of intrapartum regional analgesia		0.88 (0.81-0.96)
					Episiotomies		0.83 (0.77-0.90)
					Rates of abortion complication		1.74 (0.82-3.70)
					Adverse effects		1.15 (0.84-1.56)
					**Nurses versus doctors:**		
					Repeat consultation		0.90 (0.35-2.32)
					Better physical function		1.06 (0.97-1.15)
					Attendance to follow-up visit		1.26 (0.95-1.67)
					Attendance at emergency after receiving care		1.02 (0.87-1.14)
					Satisfaction with the care received by nurses		0.20 (0.14-0.26)

**Hatem 2008 **[47]	In midwife-led care, the midwife is the woman’s lead professional, but one or more consultations with medical staff are often part of routine practice.	RCT’s: 11	HIC		Antenatal hospitalization		0.90 (0.81-0.99)
					Regional analgesia		0.81 (0.73-0.91)
					Episiotomy		0.82 (0.77-0.88)
					Instrumental delivery		0.86 (0.78-0.96)
					Intra-partum analgesia/anesthesia		1.16 (1.05-1.29)
					SVD		1.04 (1.02-1.06)
					Feeling in control during child birth		1.74 (1.32- 2.30)
					Birth attended by midwife		7.84 (4.15-14.81)
					Initiate breast feeding		1.35 (1.03-1.76)
					Cesarean births		0.96 (0.87-1.06)
					Fetal loss before 24 weeks		0.79 (0.65-0.97)
					Fetal loss/ neonatal death at least 24 weeks		1.01 (0.67-1.53)
					Fetal / neonatal death		0.83 (0.70-1.00)
					Hospital stay		-2.00 (-2.15- -1.85)

**Laurant 2004 **[48]	Focus was on nurses working as substitutes for primary care doctors. Supplementation refers to the situation where a nurse supplements the care of the doctor by providing a new primary care service	RCT/Quasi: 13 Before After: 13	Doctors and nurses in HIC	**Nurse versus doctors**		Yes	
				Patient satisfaction			0.28 (0.21-0.34) favors nurses
				Patient recall			1.34 (1.20-1.49) favors nurses
				Prescribing rates			1.00 (0.96-1.05)
				Referral rates			0.79 (0.58-1.07)

**Lewin 2010 **[49]	Any intervention delivered by LHWs and intended to improve maternal or child health (MCH) or the management of infectious diseases.	RCT: 82	LHW’s majority in LMIC		Immunization uptake		1.22 (1.10-1.37)
					Initiation of breastfeeding		1.36 (1.14 - 1.61)
					Any breastfeeding		1.24 (1.10-1.39)
					Exclusive breastfeeding		2.78 (1.74- 4.44)
					TB cure rates		1.22 (1.13 - 1.31)
					TB preventive treatment completion		1.00 (0.92 - 1.09)
					Child morbidity		0.86 (0.75-0.99)
					Child mortality		0.75 (0.55-1.03)
					Neonatal mortality		0.76 (0.57-1.02)
					Care seeking for childhood illness		1.33 (0.86-2.05)

**Pyone 2012 **[41]	Training of GP’s and assistants o perform caesarean sections	Studies: 03	Assistant medical officers, GP		Maternal health outcomes, staff retention	No	Narrative

**Thompson 2003 **[50]	Interventions included dietary advice given by a dietician or a nutritionist compared with another health professional (e.g. doctor or nurse) or self-help resources.	RCT’s: 12	Dietitians , health professionals, nurses, doctors in HIC	**Dieticians vs. Dr.**		Yes	-0.25 mmol/L (-0.37, -0.12)
				Blood Cholesterol			Favors dietician
				**Dietician vs. self help**			-0.10 mmol/L (-0.22, 0.03)
				Blood cholesterol			
				**Dietician vs. nurses**			-0.06 mmol/L (-0.11, -0.01)
				HDLc			Favors dietician
				**Dietician vs. counselor**			-5.80 (-8.91, -2.69)
				Body weight			Favors dietician

**Vieira 2012 **[42]	Included studies where Traditional Birth Attendants had been attending births prior to the intervention; and a transition to skilled health personnel were in progress or planned. The intervention was an increase in birth rate with skilled health professionals	6 studies	Skilled health personnel		Obstetric mortality ratio		OR: 0.35 (95% CI 0.13-0.93)
					Decrease in maternal deaths		OR: 0.31 (95% CI 0.11-0.81)
					Birth by a physician		Increased with ranges from 22.4% to 70.2%
					Birth by C-Section		1.67 times more likely

Care provided by midwives was found to be associated with significant improvements in antenatal hospitalization (RR: 0.90, 95% CI: 0.81-0.99), episiotomy (RR: 0.81, 95% CI: 0.77-0.88), instrumental delivery (RR: 0.86, 95% CI: 0.78-0.96), initiation of breast feeding (RR: 1.35, 95% CI: 1.03-1.76) and hospital stay (MD: -2.00,95% CI: -2.15 to -1.85) [47]. Another review evaluating the effects of CHW interventions reported significant impacts on immunization uptake (RR: 1.22, 95% CI: 1.10-1.37), breast feeding initiation (RR: 1.36, 95% CI: 1.14-1.61), child morbidity (RR: 0.86, RR: 0.75-0.99) and TB cure rates (RR: 1.22, 95% CI: 1.13-1.31) compared to routine care [49]. Care delivered by MLHW versus non-MLHW was found comparable for antenatal hospitalization, antepartum hemorrhage, fetal loss/ neonatal deaths, induction of labour, spontaneous vaginal delivery, instrumental vaginal births, cesarean sections, perineal laceration requiring suturing, post-partum hemorrhage, preterm birth, LBW and admission to neonatal special/intensive care unit. Furthermore, ANC provided by midwives alone gave comparable results on a range of MNH outcomes compared to care provided by doctors working in a team with midwives. These findings suggest that care delivered by MLHW can be safe and effective [46]. Improved patient satisfaction and recall was reported when nurses were substituted for primary health care provision in place of doctors; although the data was from HIC only [48]. In another review, dietary counseling given by dieticians was comparable to that by nurses or doctors [50].

### Training of human resources

We found eighteen [23,39,51-66] reviews on human resource training with median quality score of 8.5 on AMSTAR rating scale. Three reviews reported MNH specific outcomes including ANC, institutional delivery, cesarean-section rates-section rates, referrals, stillbirths, maternal, perinatal and neonatal mortality while other reported outcomes included knowledge, compliance, performance and patient satisfaction. Most of the reviews evaluating training programs for outreach workers, CHW, community midwives or TBA were conducted in LMIC while reviews on the training of other licensed healthcare professional like physicians, residents, fellows, and medical students were from HIC. Table 3 summarizes the characteristics of the included reviews.

**Table 3:** Characteristics of the included reviews for human resources-training

Reviews (n=18)	Description of included interventions	Type of studies included (no)	Targeted health care providers	Outcome reported		Pooled data (Y/N)	Results
						
				Other outcomes	MNCH specific outcomes		
**Bhutta 2010 **[51]	In-service training to health personnel only, defined as SBAs (nurses, midwives, doctors or health personnel with midwifery skills) for better maternal outcomes.	Before after:08, Quais:02, Cross-sectional: 2	Skilled birth attendants (doctors, nurses and midwives) as well as to other service providers (lab tech) in LMIC		Cesarean section	No	1.78 (0.34-9.32)
					Maternal mortality		0.57 (0.36-0.91)
					Obstetric complications		1.72 (0.72-4.10)
					Institutional delivery		2.92 (2.09-4.06)
					Referrals		0.57 (0.25-1.31)
					Mean antenatal visits		0.90 (0.47-1.33)

**Giguere 2012 **[72]	The distribution of published or printed recommendations for clinical care and evidence to inform practice, including clinical practice guidelines, journals and monographs.	14 RCTs 31 ITS	All health care professionals	**PEM vs. no intervention**		Yes	
				Practice outcomes: (categorical)			Median absolute risk difference 0.02 (range 0, 0.11) i.e. 2% absolute improvement
				Profession practice outcomes: (continuous)			median improvement in standardised mean difference 0.13 (range -0.16, 0.36)

**Forsetlund 2009 **[53]	We included the following types of educational meetings: conferences, lectures, workshops, seminars, symposia, and courses.	Trials: 81	Qualified health professionals or health professionals in postgraduate training mostly in HIC	**Any intervention with educational meeting vs. no intervention:**		Yes	6% (1.8-15.9)
				Compliance			
				**Only educational meeting vs. no intervention:**			
				Compliance			6% (2.9-15.3)
				Achievement of treatment goal			3 (0.1-4)

**Hulscher 2005 **[54]	Within the professional oriented interventions we distinguished between conceptually different interventions: information transfer, learning through social influence, feedback and reminders.	RCT: 37 Quasi: 18	Family physicians, general internists, gynaecologists, obstetricians, pediatricians and sometimesother professionals like nurse practitioners and radiologists in HIC	**Preventive services:**			
				Group education vs. no intervention			Range: -4% - 31%
				Multifaceted interventions versus group education			Range: -31% - 28%

**Hyde 2000 **[55]	Critical appraisal is the process of assessing and interpreting evidence by systematically considering its validity, results and relevance to an individual’s work.	RCT:01 NRCT: 08 CBA: 07	Doctors, midwives, managers and researchers	Knowledge		Yes	0.10 (0.06-0.14)
				Skills			14/16 comparisons showed positive effect
				Attitude			4/4 comparisons showed positive impact

**Lassi 2010 **[39]	Intervention packages that included additional training of outreach workers namely, female health workers/visitors, community midwives, community/village health workers, facilitators or TBAs in maternal care during pregnancy, delivery and in the postpartum period; and routine newborn care.	18 cluster-randomized/quasi-randomized trials	Outreach workers namely, female health workers/visitors, community midwives, community/village health workers, facilitators or TBAs in LMIC		Maternal mortality	Yes	0.77 (0.59-1.02)
					Maternal morbidity		0.75 (0.61-0.92)
					Neonatal mortality		0.76 (0.68-0.84)
					Perinatal mortality		0.80 (0.71-0.91)
					Referral		1.4 (1.19-1.65)
					Early breast feeding		1.94 (1.56-2.42)

**Légaré 2010 **[56]	Interventions may include but are not limited to the distribution of printed educational material, educational meetings, audit and feedback, reminders, and patient-mediated interventions	RCT’s:05	Healthcare professionals, residents, fellows, and other pre licensurehealthcare professional	**Adoption of shared decision making:**		No	
				Both patient mediated interventions			1.06 (0.62-1.5)
				Multifaceted intervention vs usual care			2.11 (1.3-2.9)

**Lugtenberg 2008 **[57]	CPGs were defined as ‘‘systematically developed statements to assist practitioner decisions about appropriate healthcare for specific clinical circumstances.’’	cRCT: 10, before after: 10, ITS: 1	Physicians	Process outcomes		No	17/19 studies showed significant improvements
				Patient outcomes			6/9 studies showed significant but small improvements

**Norman 1998 **[58]	The conscientious explicit and judicious use of current evidence in making decisions about the care of individual patients	RCT: 03 CT:06 Cohort: 01	Medical students, residents	**Undergraduate knowledge**		No	Mean gain 17.0%; [SD] 4.0%).
				Residents knowledge			Mean gain 1.3%; SD 1.7%).

**O’Brien 2007 **[59]	Educational outreach visits, defined as use of a trained person from outside the practice setting who meets with healthcare professionals in their practice settings to provide information with the intent of changing their performance.	RCT: 69	Healthcare professionals	Compliance		Yes	5.6% (3.0-9.0%)
				Prescribing			4.8% (3.0-6.5%)
				Professional Performance			6.0% (3.6-(16.0)

**Opiyo 2010 **[60]	Following in-service training courses aimed at changing provider behavior in the care of the seriously ill newborn or child: Neonatal and pediatric life support courses e.g. NLS, NRP, PALS, PLS, and others. Life support elements. Other in-service newborn and child health training courses aimed at the recognition and management of the seriously ill child	RCT: 02	Doctors (general practitioners and specialists), nurses, pharmacists and dieticians/nutritionists, in outpatient or hospital-based settings in LMIC		Performance of adequate initial resuscitation steps	No	2.45 (1.75-3.42)
					Frequency of inappropriate and potentially harmful practices		0.40 (0.13-0.66)

**Oxman 1995 **[61]	Participation of health care providers in conferences, lectures, workshops or traineeships outside their practice settings.	Trials: 17	General healthcare providers	Change in health outcome and performance		No	Narrative

**Reeves 2008 **[62]	An IPE intervention occurs when members of more than one health and/or social care profession learn interactively together, for the explicit purpose of improving inter-professional collaboration and/or the health/well being of patients/clients.	RCT: 04 CBA: 02	Health and social care professionals	Patient satisfaction		No	4/6 reported positive outcomes
				Collaborative team behavior			
				Reduction in clinical error			

**Sibley 2012 **[73]	Trained birth attendants training	RCT: 6	Trained birth attendants		**Trained birth attendants versus untrained birth attendants:**	No	**Adjusted OR (95% CI)**
					Still births		0.69 (0.57 to 0.83)
					Perinatal death		0.70 (0.59 to 0.83)
					Maternal mortality		0.74 (0.45 to 1.22)
					Referral		1.50 (1.18 to 1.90)
					Neonatal deaths		0.71 (0.61 to 0.82)
					Obstructed labor		1.26 (1.03 to 1.54)
					Hemorrhage		0.61 (0.47 to 0.79)
					Puerperal Sepsis		0.17 (0.13 to 0.23)

**Smits 2002 **[63]	Educational intervention was problem based learning	RCT’s: 06	Post graduate continuing education in HIC	Participant’s knowledge, performance, satisfaction		No	Narrative
				Patients health			
				Follow-up			

**Thomas 1999 **[64]	Effect of clinical guideline on behavior of nurses, midwives or PAM's, on patient outcomes	RCT: 13 CBA: 2 ITS: 03	Nursing, midwifery, health visiting, podiatry, speech and language therapy, physiotherapy and occupational therapy, pharmacy and radiography	General effectiveness		No	Narrative

**Wensing 1998 **[65]	Information transfer through group education, reading material and patient education	RCT: 39 CBA: 22	Physicians in HIC	Effectiveness against the reported outcome measures		No	Narrative

**Worral 1997 **[66]	Interventions to improve medical practice like dissemination strategies such as conferences or mailing	13 trials	Physicians in HIC	Conditions studies		No	5/13 studies showed statistically significant results

In LMIC settings, training TBA (for providing basic antenatal, natal and postnatal care; preventive essential newborn care, breastfeeding counseling; management and referral of sick newborns; skills development in behavior change communication and community mobilization strategies to promote birth and newborn care preparedness) as a part of community based intervention packages showed significant improvement in referrals (RR: 1.4, 95% CI: 1.19-1.65) and early breast feeding rates (RR: 1.94, 95% CI: 1.56-2.42) with significant reductions in maternal morbidity (RR: 0.75, 95% CI: 0.61-0.92), neonatal mortality (RR: 0.76 95% CI: 0.68-0.84) and perinatal mortality (RR: 0.80, 95% CI: 0.71-0.91) [39]. TBA training also reduced peri-neonatal mortality however there was insufficient data to provide the evidence base needed to establish training effectiveness [23]. In-service training courses specifically directed to improve the management of critically ill newborns showed significant improvements in performance of initial resuscitation (RR: 2.45, 95% CI: 1.75-3.42) and reduced the frequency of inappropriate and potentially harmful practices (RR: 0.40, 95% CI: 0.13-0.66) [60] while in-service trainings for skilled birth attendants (doctors, nurses and midwives) were found to be associated with significant impacts on maternal mortality (RR: 0.57, 95% CI: 0.36-0.91) and institutional delivery (RR: 2.92, 95% CI: 2.09-4.06) [51]. The impacts on obstetric complication, ceasarean sections, ANC and referrals were non-significant.

For outcomes other than MNH, educational outreach visits and meetings were associated with improved compliance (5.6%, Range: 3-9%), prescription (4.8%, Range: 3-6.5%), professional practice (6%, Range: 3.6-16%), and some of the patient healthcare outcomes [53,59]. The evidence for continuing medical education, problem based learning and clinical practice guideline implementation remained inconclusive [63,64,66]. The impact of critical appraisal teaching on physicians’ behavior was mixed with positive impacts on improving knowledge (MD: 0.10, 95% CI: 0.06-0.14), skills, and attitude [55].

### Community mobilization

We found two [39,67] reviews evaluating the impact of community mobilization strategies and formation of community support groups with median quality score of 8 on AMSTAR criteria. Both the reviews reported the impacts on MNH outcomes with one from HIC and the other from LMIC. Table 4 summarizes the characteristics of the included reviews.

**Table 4:** Characteristics of the included reviews for Community Mobilization and Support Groups

Reviews (n=02)	Description of included interventions	Type of studies included (no)	Targeted health care providers	Outcome reported		Pooled data (Y/N)	Results
						
				Other outcomes	MNCH specific outcomes		
**Jepson 2000 **[67]	Formation of a committee of community representatives, promotion of the screening service, and implementation of an appointment system by the committee	RCT: 02	All people eligible to participate in a screening program as defined by the entry criteria for that program, included population groups such as pregnant women, neonates, children and adults in HIC		Mammogram uptake	No	Range: 5%-15%

**Lassi 2010 **[39]	Intervention packages that included additional training of outreach workers namely, female health workers/visitors, community midwives, community/village health workers, facilitators or TBAs in maternal care during pregnancy, delivery and in the postpartum period; and routine newborn care.	18 cluster-randomized/quasi-randomized trials	outreach workers namely, female health workers/visitors, community midwives, community/village health workers, facilitators or TBAs in LMIC		Maternal mortality	Yes	0.77 (0.59-1.02)
					Maternal morbidity		0.75 (0.61-0.92)
					Neonatal mortality		0.76 (0.68-0.84)
					Perinatal mortality		0.80 (0.71-0.91)
					Referral		1.4 (1.19-1.65)
					Early breast feeding		1.94 (1.56-2.42)

Community based intervention packages involving family members through community support and advocacy groups and community mobilization along with additional training of outreach workers was reported as one of the most successful strategies showing significant impacts on maternal morbidity (RR: 0.75, 95% CI: 0.61-0.92), neonatal mortality (RR: 0.76 95% CI: 0.68-0.84), perinatal mortality (RR: 0.80, 95% CI: 0.71-0.91), referral (RR: 1.4, 95% CI: 1.19-1.65) and early breast feeding (RR: 1.94, 95% CI: 1.56-2.42) [39]. Another review reported increased uptake of mammogram ranging from 5% to 15% with the formation of community groups[67].

## Discussion

There is a greater body of existing evidence on the effectiveness of community based inputs for improving MNH outcomes in LMIC compared to district and facility level inputs (discussed in papers 3 and 4) [68,69]. At community level, home visitation, community mobilization, women’s support groups and training of the CHW and TBA have shown maximum impact on a range of MNH outcomes. Community based generation of funds for transportation has also shown significant impacts in resource limited settings of India and sub-Saharan Africa. Interventions delivered through midwives, CHW and MLHW have not only demonstrated comparable outcomes when compared to routine non-MLHW care delivery but also reported better results for some of the outcomes. Specialized outreach clinics, continuing medical education, problem based learning, clinical practice guideline implementation and critical appraisal showed inconclusive and mixed results.

Although the process pathways for the effectiveness of community delivered interventions are uncertain, they seem to influence community awareness, behavior change and practices, such as accessing skilled birth, use of clean delivery kits, breastfeeding and care seeking for maternal and newborn illnesses. Our overview findings greatly add to the global evidence base of intervention and delivery strategies that may improve MNH outcomes. It implies that within the community level inputs, three interventions have unparalleled significance: first, CHW who provide primary health care, can mobilize community members and impart knowledge; second, training of and linkages to TBA can provide basic prenatal and obstetric care, as well as referrals where skilled birth attendants are absent; third, community support groups, especially women’s groups, can empower communities and help in problem solving and planning to improve opportunities for women’s health, as well as care for mothers and newborns.

Countries in Asia and sub-Saharan Africa are facing critical shortages of healthcare workers despite of bearing 25% of the world’s diseases burden [20]. Reasons behind the migration of professional healthcare force to richer countries is suggested to be lack of incentive, poor working conditions and fewer opportunities for promotions [70]. There is also an existing pull from HIC to recruit health workers from LMIC. Given the shortage of care providers and functional health facilities, and the deeply entrenched practices, there is much interest in community-based interventions and strategies for care. Increasing the number of skilled health workers, training and educating them, providing them with incentives and improving the infrastructure is what needs to be done in all the LMIC to make progress towards achieving the Millennium Development Goals (MDG) 4 and 5.The findings from this overview testifies that increasing the availability and training of the skilled health workers including TBA and CHW for adequately recognizing and managing obstetric complications can significantly reduce maternal and neonatal morbidity and mortality especially in the resource limited settings of Asia and Africa where the highest maternal mortality burden exists with limited resources to mobilize. The challenge is to incentivize these programs and link them with formal health systems to increase retention. In many countries CHW are not linked to national health systems and are expected to work as volunteers which is a major drawback. Another existing challenge is the variation in prerequisites, recruitment, training, supervision and workload of various cadres of community workers including CHW, TBA and midwives. There is a need to streamline their functioning and delegate the activities to achieve efficient implementation and maximum impact.

With the established effectiveness of task shifting and training of CHW, future studies should focus on the factors affecting the sustainability and cost effectiveness of these interventions when scaled up [46]. There is a dearth of information on costs and equity aspects of community based programs as only a few studies have reported the actual costs incurred for saving lives or averting deaths with the use of these strategies. Researchers should now facilitate cost-effectiveness studies and consequent meta-analysis by collecting and reporting cost effectiveness data in a standardized format [39]. Further work is also needed on nurse- based care delivery models including length and frequency of contact, type of approach (e.g. individual or group, behavioral therapy or instructional techniques), level of training of practitioner, patient satisfaction and initial characteristics of patients to establish equivalence in care with the physician- based model and also for program replication [50]. Formal monitoring and evaluation of programs especially referral interventions are also necessary to develop better understanding of how referral interventions work. There is lack of data to establish effectiveness of mass media campaigns and community education as single strategies.

Outreach services may confer the most benefit to access and health outcomes in rural and underprivileged settings hence there is a need for good comparative studies in resource deprived settings rather than in HIC [33]. Among the outreach workers in the LMIC, the role of TBA is pivotal as they remain a major maternity care provider and their services expand from birth attendance to include newborn and post natal care like bathing and massage, domestic chores, and provision of care during postnatal period. Despite of that, TBA training remains controversial in relation to the global 'Safe Motherhood Initiative' as there is insufficient data to provide the evidence base needed to establish training effectiveness [23]. Hence methodologically rigorous evaluations with an adequate sample size are needed to measure the magnitude of the impact of TBA training on maternal and neonatal mortality.

Community based interventions have promising potential to provide range of services throughout the continuum of care and also reach the hard to reach population groups. Current evidence emphasizes that effective community based strategies exist to deliver a range of preventive and promotive interventions to improve the quality of care delivered for MNH and many of these interventions have the potential to reduce maternal, perinatal and neonatal morbidity and mortality. There is now a need to implement them on a larger scale throughout the LMIC. These interventions exist within the current health systems in most of the LMIC and hence policies are needed to integrate and sustain various task shifting and training interventions with the maternal health programs within their health systems. All stakeholders including governments, communities and donors need to work together to form these policies and develop models of health care to suit the needs of their own population. Still more work needs to be done in areas of recruitment, deployment and retention of the community based health care workers in the rural and underprivileged areas and improve the working conditions for them.

## List of abbreviations used

ANC: Antenatal Care; AMSTAR: Assessment of Multiple Systematic Reviews; CHW: Community Health Workers; CI: Confidence Interval; ENC: Emergency Newborn Care; HIC: High Income Country; LBW: Low Birth Weight; LHV: Lady Health Visitor; LHW: Lay Health Workers; LMIC: Low and Middle Income Country; MD: Mean Difference; MLHW: Mid Level Healthcare Worker; MNH: Maternal Newborn Health; NLS: Newborn life Support; NRP: Neonatal Resuscitation Programs; PNC: Postnatal Care; RD: Risk Difference; RR: Relative Risk; SBA: Skilled Birth Attendant; TBA: Traditional Birth Attendant

## Competing interests

We do not have any financial or non-financial competing interests for this review.

## Author contributions

All authors contributed to the process and writing of the manuscript.

## Peer review

Peer review reports are included in Additional file 1.

## Supplementary Material

Additional file 1 Click here for file

## Declarations

This article has been published as part of *Reproductive Health* Volume 11 Supplement 2, 2014: Quality of Care in Maternal and Child Health. The full contents of the supplement are available online at http://www.reproductive-health-journal.com/supplements/11/S2.
