# Supplementary material for: Evidence from community level inputs to improve quality of care for maternal and newborn health: interventions and findings
Source: Reprod Health. 2014 Sep 4;11(Suppl 2):S2. doi: 10.1186/1742-4755-11-S2-S2 (PMC4160921; doi:10.1186/1742-4755-11-S2-S2)
Supplement: Additional file 1 [file 1742-4755-11-S2-S2-S1.pdf]

Referee's comments to the authors– this sheet WILL be seen by the author(s) and published alongside the article

Article ref no.	
Title	Evidence from community level inputs to improve quality maternal and newborn health: Interventions and findings
Author(s)	Zohra S lassi; Jai K. Das; Rehana A Salam & Zulfiqar A Bhutta
Referee's name	J. Shea

When assessing the work, please consider the following points, where applicable:

[USE THE APPROPRIATE QUESTIONS FOR THE ARTICLE TYPE TO BE REVIEWED – SEE Reviewer Guidelines above]

1. Is the question posed by the authors new and well defined?
2. Are the methods appropriate and well described, and are sufficient details provided to replicate the work?
3. Are the data sound and well controlled?
4. Does the manuscript adhere to the relevant standards for reporting and data deposition?
5. Are the discussion and conclusions well balanced and adequately supported by the data?
6. Do the title and abstract accurately convey what has been found?
7. Is the writing acceptable?

Please make your report as constructive and detailed as possible in your comments so that authors have the opportunity to overcome any serious deficiencies that you find and please also divide your comments into the following categories:

- Major Compulsory Revisions (which the author must respond to before a decision on publication can be reached)
- Minor Essential Revisions (such as missing labels on figures, or the wrong use of a term, which the author can be trusted to correct)
- **Discretionary Revisions (which are recommendations for improvement but which the author can choose to ignore)**

Where possible please supply references to substantiate your comments.

When referring to the manuscript please provide specific page and paragraph citations where appropriate.

The content of the review is relevant and important.

Abstract: The question/objective is not articulated

Background: The first sentence is grammatically incorrect. The second paragraph can be improved (see Paper 4 in this series).

Community Level Characteristics: Each of the categories identified will benefit from revision. For example: The Outreach and Home Visitation section needs an introductory

sentence, followed by a concise, succinct description of the features that comprise Outreach and home visitation. The last sentence of this section needs revision.
The *Task Shifting* definition provided is clumsy.
The *Human Resource Training* section should have a lead-in sentence that foregrounds the critical and chronic health workforce shortage and deficiency in essential skills.
Community mobilization and Support Groups: This section does not adequately explain the purpose of community engagement or the role of community support groups.

Search Strategy: The preliminary search strategy included all available systematic reviews on pre-defined community level interventions as presented in the conceptual framework (presented in paper1). It seems that the initial search included a number of related but not relevant papers and these were excluded. The search strategy is generally well described, but I would advise that the authors be more specific about what keywords and what combination (strings) they searched for. It is important to note that Cochrane Systematic Reviews include only Randomised Controlled Trials (RCTs) and do not include a wide variety of other research designs, like mixed method studies. Social science and policy reviews may have been excluded from this review, and these are often excellent sources of quality of care research. The Pubmed search and “reviews that met the inclusion criteria” need to be described. General information about the study designs included in reviews other than Cochrane Systematic Reviews is recommended.

Findings: A strength in this section is the description of commonly reported outcomes.
A shortcoming in the Training Human Resources section is that it does not describe the variations in TBA prerequisites, recruitment, training, supervision and workload. The same applies to LHWs.

The authors should consider replacing the term “disadvantaged” settings to under resourced or underserved settings.

Discussion:
Community based interventions were most widely assessed for their effectiveness in improving MNH outcomes in LMIC settings compared to district and facility level inputs.- sentence meaning is unclear.
Variations in TBA/LHW/CHW prerequisites, recruitment, training, supervision and workload must be described if not done earlier in the manuscript.
In the discussion it is advisable that the authors describe the conclusion drawn from the reviews, then provide supporting evidence. For example: The challenge is to incentivize these programs to increase retentionas CHWs in majority of the programs are not linked to the formal health system of the country and expected to work as volunteers, which is a major drawback. Conversely, in Pakistan, LHWs are considered civil servants and given one-year contracts, hence retention rate is high in these areas with the dropout rates of less than 1 per cent per year(57).- (p.8). The point is not expressed well so the association between the lack of formal employment procedures and poor retention is unclear, even though the last sentence appears to provide some explanation. In fact the entire paragraph is not systematic and needs revision. The Discussion is not effectively linked to the Findings; the flow is not smooth, leaving the reader to look for the associations between the two sections.

In the section that appears to be the Conclusion, the first sentence is difficult to understand: Community based interventions have promising potential to provide care throughout the

continuum of maternal and newborn health as community based platforms provide access to reproductive health care, care during pregnancy and childbirth along with the basic newborn care to the hard to reach groups (p.9).
"Governments, stakeholders and donors need to work together ...". This sentence would be better if it read: "All stakeholders, including governments, donors etc ...."

A systematic review conducted by Lassi et al states:" The results of our meta-analysis indicate that antenatal care provided by midwives alone gave comparable results on most outcome measures to care provided by doctors working in a team with midwives" (p. 828). This is a significant finding for Task Shifting as discussed in this paper.

Reference: Lassi, Z. S., Cometto, G., Huicho, L., & Bhutta, Z. A. (2013). Quality of care provided by mid-level health workers: systematic review and meta-analysis. *Bulletin Of The World Health Organization*, *91*(11), 824-833I. doi:10.2471/BLT.13.118786

In this manuscript there are several language/editing issues. For example:

- "Community-based interventions to improve MNH" – when an abbreviation is used for the first time in the paper it must be written out in full; thereafter it can be used throughout the paper (p.3).
- Sentences are too long: They focus on resources such as local knowledge, volunteers' time, community confidence and trust as channels for delivery of a full spectrum of promotive, preventive, and curative interventions including provision of basic antenatal, natal and postnatal care, preventive essential newborn care, breastfeeding counseling; management and referral of sick newborns; skills development in behavior change communication and community mobilization strategies to promote birth and newborn care preparedness (p.3).
- These programs although do not substitute.....
- For this review, we have broadly categorized these interventions into; outreach services – the semi-colon should be a : (p.3).
- Task shifting for human resource management involves healthcare workers that are lesser trained than specialized personnel but can substitute for them or perform some aspects of their tasks. A range of both skilled and semi-skilled health workers can play a major role in MNH service delivery (p.3).
- " ... care provider and their expands from birth attendance to include newborn and post natal care like bathing and massage ..." – I am unclear about what this means
- According to the World Health Organization (WHO) there is a shortage of 4.25 million health workers globally and developing countries are facing the heft of this burden (p.8).
- Hence methodologically rigorous evaluations with an adequate sample size are needed to measuring the magnitude of the impact of TBA training on maternal mortality.
- Throughout the document the word "the" is inappropriately used. For example: neonatal morbidity and mortality especially in the resource limited settings of Asia and Africa (p.8)
- The reasons behind the migration of professional healthcare force to richer countries is suggested to be lack of incentive, poor working conditions and fewer opportunities for promotions (56) thus increasing the number of skilled health workers, training and

educating them, providing them with incentives and improving the infrastructure is what needs to be done in all the developing countries to make progress towards achieving the MDG 4 and 5 (p. 8) – This sentence is too long and should be broken up into 2 sentences.

The manuscript needs complete language/editing revision.

Referee's comments to the authors– this sheet WILL be seen by the author(s) and published alongside the article

Article ref no.	
Title	Evidence from community level inputs to improve quality of maternal and newborn health: Interventions and Findings
Author(s)	Zohra S Lassi[1], Jai K Das[1], Rehana A Salam[1], Zulfiqar A Bhutta[12]
Referee's name	Viva Combs Thorsen

When assessing the work, please consider the following points, where applicable:

[USE THE APPROPRIATE QUESTIONS FOR THE ARTICLE TYPE TO BE REVIEWED – SEE Reviewer Guidelines above]

8. Is the question posed by the authors new and well defined?
9. Are the methods appropriate and well described, and are sufficient details provided to replicate the work?
10. Are the data sound and well controlled?
11. Does the manuscript adhere to the relevant standards for reporting and data deposition?
12. Are the discussion and conclusions well balanced and adequately supported by the data?
13. Do the title and abstract accurately convey what has been found?
14. Is the writing acceptable?

Please make your report as constructive and detailed as possible in your comments so that authors have the opportunity to overcome any serious deficiencies that you find and please also divide your comments into the following categories:

- Major Compulsory Revisions (which the author must respond to before a decision on publication can be reached)
- Minor Essential Revisions (such as missing labels on figures, or the wrong use of a term, which the author can be trusted to correct)
- Discretionary Revisions (which are recommendations for improvement but which the author can choose to ignore)

Where possible please supply references to substantiate your comments.

When referring to the manuscript please provide specific page and paragraph citations where appropriate.

General comments:

Thank you for the opportunity to review this manuscript. Quality of care in MNH at the community level is an important topic, particularly as the deadline for the MDGs draws near. The summary of systematic reviews is extensive; however, there are issues the authors must address before a decision to accept the manuscript for publication can be made. Please see below.

Major compulsory revisions:

- **Background:** the authors should include some background information on the quality of care, what that means, at the community level. The first reference is out dated; there are more relevant and recent ones that should be used. The authors provide a laundry list of causes of deaths, but only include one reference (#2); please provide more. In the following sentence, the authors mention “early recognition”, of what and by whom? The first sentence of the second paragraph needs rewording and should focus more on justifying the focus on the community level (e.g. something to the effect that’s stated under “Community Mobilization and Support Groups” on page 4). In that same sentence the authors mention “reduce inequities”, in what?

- **Task Shifting:** Right now it reads in general terms. Providing an example or two of task shifting related to MNH care services would focus it to the subject at hand.

- **Home Resource Training:** Right now the focus is only on newborns. Add a few examples of life-saving skills for pregnant/laboring women/new mothers.
- **Community Mobilization and Support Groups:** The authors mention community participation, but have not shared examples of what community mobilization or support groups look like. The authors need to elaborate on this section.

- **Methods:** Though this manuscript is part of a series, it should still stand alone. The methods section, therefore, needs much more details; for example, for what time period does the search/review cover. Who extracted the data and how were they trained (what did the training entail); what does AMSTAR entail (and what are cut-off scores); why are the authors reporting on general health outcomes when the focus should be solely on MNH outcomes? An appendix that includes the list of pre-identified MeSH and key search terms would help the readers judge rigor and assess bias. The authors state that they “aim to systematically analyze...reviews...” (Page 4 above Panel 1), but did not provide any details in the method section on how this was done. As it all reads now, it is only descriptive.

- **Findings:** The authors identified 310 potentially relevant review titles then ended up with 42 eligible reviews. To promote more transparency and reduce publication bias, provide more details as to what was excluded and on the figure it would be helpful to possible group the excluded ones into categories. In the last paragraph of the Outreach Service section, the last variable “accesses” what were the results? In the Task Shifting section authors mention that care delivered by MLHW vs. non MLHW “reported comparable results hence suggesting that the care delivered by MLHW can be safe and effective” What were the results (what type of outcomes were the review evaluating)? The inclusion of the dietary counseling review is confusing because it is not related to maternal or newborn health care service delivery (or is it)? It’s about reducing cholesterol. Why is it included? The last sentence of this section (Task shifting) is also confusing (first, it’s an interpretation so should be moved to the discussion section, but most importantly I don’t see the connection). The last paragraph in the Training of Human Resources section (page 7) talks about “outcomes other than MNH”. It should be deleted altogether since the focus is on MNH outcomes and quality of care. In the second paragraph of the Community Mobilization section (page 8) the authors state that community-based intervention packages were reported as one of the most successful strategies and provided some statistics. Again, it would be helpful/insightful to know what in fact those packages entailed (e.g. chief-mandated taxi assistance, etc.).

- **Discussion:** The discussion tended to repeat what had already been said, instead of critically thinking about the issue and providing a deeper, more profound understanding of the issues. It lacked explanations of results that were observed (e.g. the non-significant impacts that were observed, why do the authors think that is). Were there any findings that were surprising/unexpected, etc? Why are the results of import, what are the implications?

Subheadings may help the reader (e.g. similar to the findings section, recommendations, future research, and conclusions). What are the limitations and strengths of this study? A discussion about the AMSTAR scores would also be useful (what are their implications?).

- **Tables:** I question the inclusion of some of the studies/their relevance: Table 1: Ciliska 01 (18), Elkan 00 (19), Thompson 2003 (37), Laurant 2004 (35) is that for MNH or nursing care in general?; and Table 4 Jepson 2000 (55).

- **English editing is required.**

Minor essential revisions:

- Consistency in terminology: sometimes the authors use LMIC or poor countries; trained vs. skilled attendants; home-based vs. community level
- References: document numbers missing (Ref #s 11, 12, 24); strange symbols included (Ref #s 25 & 29); check page number for Ref #19. Thoroughly check the references. Also, citations within the text—sometimes the reference number comes before the period and other times it comes after. Comply with instructions and be consistent.
- Spell out abbreviations in the abstract
- Comply with number and spelling of number conventions
- Consistency in what's included in the tables, e.g. sometimes the authors provide details about the type of studies included (in a few of them they don't); the same with the target group (i.e., whether they involve LMIC or HIC)

Discretionary revisions:

- The title: is it not a matter of improving not only the quality of maternal and newborn health but also the "care" provided? Should that be reflected in the title (both health outcomes and quality of care)?
- The length of the abstract (450+ words) seems a bit long. Does the length comply with the guidelines?
- In the background section of the abstract, the authors may consider adding a sentence shows community level intervention effective impact on maternal and newborn health outcomes.
- Panel 1 on pages four and five is somewhat redundant to what had been stated/provided in the text prior to the panel. Therefore, it is not necessary (there's no added value).
